# Adaptations of Gram-Negative and Gram-Positive Probiotic Bacteria in Engineered Living Materials

**DOI:** 10.1021/acsbiomaterials.5c00325

**Published:** 2025-05-13

**Authors:** Varun Sai Tadimarri, Tanya Amit Tyagi, Cao Nguyen Duong, Sari Rasheed, Rolf Müller, Shrikrishnan Sankaran

**Affiliations:** 1 INM - Leibniz Institute for New Materials, Saarland University, Campus D2 2, Saarbrücken 66123, Germany; 2 Helmholtz Institute for Pharmaceutical Research Saarland (HIPS), Helmholtz Center for Infection Research (HZI), Saarland University, Campus E8 1, Saarbrücken 66123, Germany; 3 German Centre for Infection Research (DZIF), Partner Site Hannover, Braunschweig 38124, Germany; 4 Saarland University, Saarbrücken 66123, Germany

**Keywords:** engineered living materials, spatial confinement, bacterial physiology, microbial behavior, engineered living materials

## Abstract

Encapsulation of microbes in natural or synthetic matrices is a key aspect of engineered living materials, although the influence of such confinement on microbial behavior is poorly understood. A few recent studies have shown that the spatial confinement and mechanical properties of the encapsulating material significantly influence microbial behavior, including growth, metabolism, and gene expression. However, comparative studies within different bacterial species under identical confinement conditions are limited. In this study, Gram-negative Escherichia coli Nissle 1917 and Gram-positive Lactiplantibacillus plantarum WCFS1 were encapsulated in hydrogel matrices, and their growth, metabolic activity, and recombinant gene expression were examined under varying degrees of hydrogel stiffness, achieved by adjusting the polymer concentration and chemical cross-linking. Both bacteria grow from single cells into confined colonies, but more interestingly, in E. coli gels, mechanical properties influenced colony growth, size, and morphology, whereas this did not occur in L. plantarum gels. However, with both bacteria, increased matrix stiffness led to higher levels of recombinant protein production within the colonies. By measuring metabolic heat from the bacterial gels using the isothermal microcalorimetry technique, it was inferred that E. coli adapts to the mechanical restrictions through multiple metabolic transitions and is significantly affected by the different hydrogel properties. Contrastingly, both of these aspects were not observed with L. plantarum. These results revealed that despite both bacteria being gut-adapted probiotics with similar geometries, mechanical confinement affects them considerably differently. The weaker influence of matrix stiffness on L. plantarum is attributed to its slower growth and thicker cell wall, possibly enabling the generation of higher turgor pressures to overcome restrictive forces under confinement. By providing fundamental insights into the interplay between mechanical forces and bacterial physiology, this work advances our understanding of how matrix properties shape bacterial behavior. The implications of these findings will aid the design of engineered living materials for therapeutic applications.

## Graphical abstract

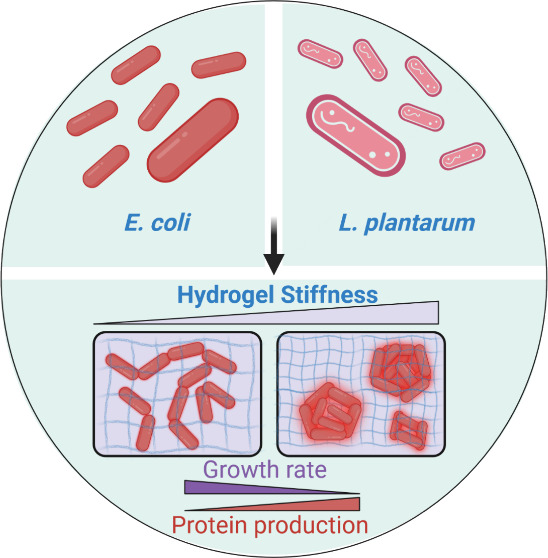

## Introduction

The interdisciplinary field of Engineered Living Materials (ELMs) has experienced significant growth in recent years, combining concepts and techniques from the fields of synthetic biology and materials science. Several proof-of-concept studies have displayed the adaptability of ELMs, demonstrating their broad-spectrum applicability in diverse sectors from construction to biomedicine.^1,2^ A common feature shared by several ELMs is the mechanical confinement of microorganisms within the materials. Confinement of microbes within different materials can be done either as single cell encapsulation^3–5^ or matrix encapsulation.^6–8^ While single cell encapsulation enables high-throughput screening of bacterial cells under confined conditions, matrix encapsulation enables the study of bacterial behavior in a relatively more natural setting that mimics the biofilm microenvironment. It enables the study of both population- and colony-level behaviors of the encapsulated bacteria. Recent studies have shown that encapsulating microbes in the hydrogel matrix can affect their growth and metabolism. This confinement-induced alteration of microbial behavior may influence the overall performance and functionality of the ELM.^9–14^ Common observations of these studies are that microbes within hydrogels grow as dense colonies that may or may not be restricted in size depending on the mechanical properties of the surrounding matrix and the availability of nutrients. Moreover, as the depth of the hydrogel increases, the diffusion of essential nutrients and gases (such as oxygen and carbon dioxide) becomes limited.^13,15^ Such limitations in resources lead to a variation in bacterial colony size, where microorganisms situated deeper within the hydrogel matrix tend to be smaller in size compared to those closer to the surface.^12,13,16,17^ Furthermore, mechanical confinement has been shown to enhance the expression of genes associated with the antibiotic resistance^17^ and maintain particular phenotypic states in microbes like yeast and cyanobacteria.^12,18^ Our previous studies have shown that encapsulating an Escherichia coli strain (ClearColi) in Pluronic F127 diacrylate (PluDA) hydrogels affected the growth rate, morphology, and protein expression of bacteria. More importantly, by modulating the degree of covalent cross-linking in these hydrogels, we observed that bacterial parameters were directly influenced by the network’s mechanical properties. Increasing degrees of covalent cross-linking led to a decrease in growth rate and colony size and an increase in colony sphericity. Surprisingly, protein production rates exhibited a nonmonotonic response, peaking at intermediate degrees of covalent cross-linking. This suggested that under these conditions, growth was sufficiently slowed to metabolically favor protein production. At the highest degree of covalent cross-linking, lower production rates result from bacteria allocating resources to overcome constrictive mechanical forces. These findings highlighted the need to optimize mechanical properties of the encapsulating material for optimal performance of an ELM. Additionally, this study involving two phylogenetically distant species unsurprisingly reveals that different microbes show different responses to confinement. However, since each study involves a single microbial species and different hydrogel components, it cannot be determined how the same mechanical confinement conditions will influence different microbes. While some studies have investigated coculture ELMs with different microbial species encapsulated in hydrogels and performing symbiotic functions, these findings have not compared the influence of confinement on the individual species.^19–22^ Investigating the effects of mechanical confinement on different microbial species will enable us to uncover species-specific adaptations and interactions within ELMs and aid in finding optimal material–microbe combinations for different applications.

In this perspective, this study compares the effect of mechanical confinement on two distinct bacterial species: Gram-negative E. coli^23^ and the Gram-positive Lactiplantibacillus plantarum.^24,25^ Both organisms are rod-shaped and exhibit the ability to survive and grow in both aerobic and anaerobic environments. E. coli is the predominant bacterial species utilized in ELM research, while L. plantarum is a widely studied probiotic strain with beneficial properties for humans, animals, and plants.^26–28^ L. plantarum also has significant industrial use in food fermentation and lactic acid production.^29,30^ In this study, we specifically chose the probiotic strains E. coli Nissle 1917 and L. plantarum WCFS1, which are genetically tractable and are of considerable interest for the development of ELMs toward biomedical applications. Both these bacterial species have been found predominantly in different parts of the human body and form biofilms with heterogeneous and dynamic mechanical properties influenced by their local environments. E. coli Nissle 1917, a nonpathogenic commensal strain, predominantly resides in the colon where it is subjected to shear forces from peristaltic movements, varying osmotic conditions, and interactions with host microbiome.^31^ In these conditions, E. coli Nissle has been reported to demonstrate robust biofilm formation that enhances mechanical resilience and promotes adhesion to epithelial surfaces.^32^ On the other hand, L. plantarum WCFS1 is predominantly found in the oral cavity and small intestine, where it is exposed to dynamic mechanical stresses such as mucosal flow, peristalsis, and chewing. It possesses a thick peptidoglycan layer and teichoic acids in its cell wall that help it withstand turgor pressure and osmotic fluctuations.^33^ Additionally, L. plantarum can modify its cell envelope and produce exopolysaccharides that contribute to biofilm matrix integrity.^34^ While nearly no quantified data exist on the mechanical properties of the natural biofilms of these strains, they can be expected to experience confined microenvironments with storage moduli ranging from 0.1 to 100 kPa as has been previously reported for different biofilms.^35–38^ Despite their similarities, the strains exhibit significant differences in their cellular architecture and physiology, including, cell-wall composition, cell membranes, cytoplasmic turgor pressure, and metabolic pathways.^39–41^ E. coli is metabolically versatile with efficient responses to nutrient shifts, whereas L. plantarum has a highly adaptable genome that supports rapid adaptation to acid stress and variable oxygen levels.^42,43^ Transcriptomic analysis of encapsulated bacteria (Gram-positive: Staphylococcus aureus and Gram-negative: E. coli) has shown that the rigidity of the encapsulated matrix plays a key role in mediating the dynamics of bacterial colony growth in the mechanically constrained state and affects the metabolic processes within the bacteria leading to antibiotic resistance.^17^

Given these distinct cellular characteristics, this study aimed to explore how E. coli Nissle 1917 and L. plantarum WCFS1 respond differently to mechanical confinement. To create the different mechanical conditions, we employed PluDA hydrogels, expanding upon our previous research by varying both the degree of chemical cross-linking and polymer concentration, resulting in nine distinct experimental conditions. The bacterial responses were assessed in terms of colony growth, morphology, protein production (confocal microscopy), and metabolic activity (isothermal microcalorimetry). These analyses suggested that probiotic E. coli and L. plantarum share some common responses to mechanical confinement but are likely to employ distinct mechanisms to grow and maintain activity under spatial constraints.

## Experimental Section

### Preparation of Precursor Solutions

Pluronic diacrylate (PluDA) was synthesized by the reaction of Pluronic F127 (Plu) (Sigma-Aldrich, P2443) with acryloyl chloride (Sigma-Aldrich, A24109) in the presence of triethylamine according to a previously reported protocol^44^ to achieve a functionalization degree of 90%. The 23% (w/w) Plu and PluDA stock solutions were prepared separately in two different nutrient broths: LB medium (Lysogeny broth) (Carl Roth) (for Escherichia coli Nissle 1917 experiments) and MRS medium (de Man–Rogosa–Sharpe) (Carl Roth) (for Lactiplantibacillus plantarum WCFS1 experiments). All the solutions were supplemented with the Irgacure 2959 photo initiator (2-hydroxy-4-(2-hydroxyethoxy)-2-methylpropiophenone, from Sigma-Aldrich) (0.2% w/v) to facilitate cross-linking when exposed to UV radiation. The 16.7% and 20% solutions of Plu and PluDA were prepared by diluting 23% stock solutions with the respective solvent (LB or MRS medium). All of the solutions were stored at 4 °C until use.

### Rheology of Polymer Combinations

A rotational rheometer (DHR3, TA Instruments) was used to measure the rheological properties. A 20 mm Peltier plate (serial number 106155, transparent to UV) was used as a bottom plate, and a 12 mm stainless steel disk was used as a top plate. Experiments were performed at room temperature (25 °C), and a UV source (Omnicure, Series 1500, 365 nm, 6 mW/cm^2^) was mounted on the rheometer. A gap of 300 μm between the plates and a volume of the precursor polymer solution of 35 μL were used for the experiment. After the 35 μL of sample was pipetted on the bottom plate, 2 min of UV exposure was programmed followed by test measurements after 10 min of pipetting the sample. Strain sweeps were conducted from 0.1 to 1000% at a frequency of 1 Hz. From these results, a linear viscoelastic region was identified, and the storage modulus per polymer condition was plotted using data obtained from the plots.

### Genetic Modification of Bacterial Strains and Culturing the Bacteria

Escherichia coli Nissle 1917 and Lactiplantibacillus plantarum WCFS1 strains were engineered with p256-mCherry plasmids to obtain the fluorescent constructs of the respective strains. E. coli Nissle was transformed with p256-mCherry with the kanamycin selection marker, and L. plantarum WCFS1 was transformed with p256-mCherry with the erythromycin selection marker. Both engineered strains express mCherry intracellularly within the bacteria, which helps in tracking their growth and behavior using fluorescence microscopy. Competent cells of both strains and transformation were performed using the published protocols.^45–47^

Respective colonies of both strains were inoculated either from an agar plate or from their glycerol stocks. E. coli Nissle 1917 was grown in the LB medium supplemented with kanamycin antibiotic (50 μg/mL), whereas L. plantarum was grown in the MRS medium supplemented with erythromycin (10 μg/mL) for 14–16 h at 37 °C followed by subculturing the bacteria on the next day to an OD_600_ (optical density at 600 nm) of 0.1 and letting it grow until the log phase (0.5–0.8) before encapsulation.

### Microscopy of Bacterial Culture

Grown cultures of Escherichia coli Nissle 1917 and Lactiplantibacillus plantarum WCFS1 strains expressing mCherry were concentrated to OD 1, and 200 μL of diluted cultures was pipetted into a 96-well black plate with a transparent bottom. Fluorescence microscopy of these bacterial cultures was performed using a table-top Keyence microscope (BZ-X800E). Multichannel images were captured at 100× magnification using the CH3 (red fluorescence) and CH4 (brightfield) channels, and the images were analyzed using ImageJ.

### Live/Dead Staining Assay

A live/dead bacterial viability kit (Thermo Fisher scientific, Germany) was used to assess the viability of bacteria after UV exposure. Overnight grown bacterial culture was subcultured and grown up to an OD_600_ of 1. The bacterial culture was spun down and washed twice with 1× PBS. The bacterial pellet was resuspended in 1 mL of PBS. Premixed solutions (1.5 μL each) of SYTO-9 (binds to live cells) and propidium iodide (binds to dead cells) were added to the resuspended bacterial pellet and incubated at 37 °C for 1 h followed by three washes with PBS. The bacterial pellet was resuspended in 1 mL of PBS and was used for imaging the stained bacteria. The resuspended solution was diluted by a 10^4^ dilution factor in PBS to attain the samples for analysis using a flow cytometer. The positive control for the experiments was untreated live bacteria.

### Flow Cytometry

The fluorescence intensity of stained bacterial solutions was measured using the Guava EasyCyte BG flow cytometer (Luminex, USA). A predesigned gate based on forward-side scatter and side scatter thresholds was used to filter out the background noise from the analysis. A presaved program to measure red and green fluorescence was used to quantify the live/dead bacterial populations in the sample, and 5000 bacteria events were recorded. The Luminex Guavasoft 4.0 software for EasyCyte was used for analyzing data.

### Bacterial Encapsulation for Microscopy

#### Preparation of the Polymer Mix

Fifteen, 18, and 21% w/w solutions of pluronic F-127 (Plu) and pluronic F-127 diacrylate (PluDA) were prepared in LB and MRS media, respectively. The pure pluronic F-127-based polymer solution is referred to as DA0, and only pluronic F-127 diacrylate-based polymer solution is referred to as DA100. Equal volumes of DA0 and DA100 were mixed and vortexed in an Eppendorf instrument to prepare the DA50 solution comprising equal parts of physically cross-linkable pluronic (DA0) and chemically cross-linkable pluronic diacrylate (DA100).

#### Encapsulation Protocol

Overnight incubated cultures of E. coli Nissle 1917 and L. plantarum WCFS1, both expressing the mCherry fluorescent protein, were stopped at an optical density (OD_600_) of 0.6–1 and suspended in a specific composition of polymer precursor solutions (at 4 °C) in the volume ratio of 9:1 (polymer solution/bacterial culture) to achieve a final OD_600_ of 0.01 (E. coli Nissle: 1 × 10^4^ CFU; L. plantarum: 6 × 10^3^ CFU) within the gels having final (w/w) polymer concentrations of 15, 18, and 21%. This mix was then vortexed immediately before being placed on ice to ensure that the pluronic solutions remain in their liquid state. Ten microliters of the polymer-bacteria suspension was pipetted into the well of an Ibidi μ-Plate 96 Well. Once all wells were pipetted with respective samples, the plate was transferred to a UV chamber and exposed to UV radiation (365 nm) for 2 min at an intensity of 6 μW/cm^2^ (the effect of UV irradiation on bacterial viability has been tested using live/dead staining assay, and no significant effects on viability were found; Figure S4a,b). Thirty microliters of silicone oil (350 cSt, Sigma-Aldrich) was pipetted in all wells with polymerized gels to prevent the drying of the hydrogel during the experiment. The plate was incubated at 37 °C until it was transferred to microscopy analysis.

### Kinetic Microplate Reader Measurements to Study Bacterial Growth and Recombinant Protein Production Rates

Engineered E. coli Nissle and L. plantarum were encapsulated in different Plu/PluDA formulations, as mentioned in the section above. Cross-linked bacterial gels of 10 μL volume were fabricated in an Ibidi μ-Plate 96 Well covered by 30 μL of silicone oil. The plate was covered, and an overnight growth kinetics measurement was set using the microplate reader Infinite 200 Pro (Tecan Deutschland GmbH, Germany) for a duration of 24 h (one measurement every hour) to measure the OD_600_ and fluorescence intensity of mCherry expression (ex/em: 587/625 nm) from the bacterial hydrogels with a gain of 100 and *Z*-position of 21,760 μm. The obtained raw data were analyzed using GraphPad Prism to plot the growth rate and fluorescence intensity plots using the formulas^25^ below:

$$\mathrm{growth}\;\mathrm{rate}=({\mathrm{OD}}_{600}\;\mathrm{at}\;T2-{\mathrm{OD}}_{600}\;\mathrm{at}\;T1)/\Delta T$$ 1

fluorescenceintensityrate=(fluorescenceunitsatT2−fluorescenceunitsatT1)/ΔT 2

### Image Acquisition and Data Analysis for Colony Volume, Colony Sphericity, and Fluorescence Intensity

Samples were imaged using a Zeiss Cell Discoverer 7 microscope equipped with a ZEISS LSM 900 and an AiryScan 2 (Zeiss, Oberkochen, Germany). Fast AiryScan mode was used with a Zeiss Plan-Apochromat 20×/0.95 objective and a 1× tube lens (Optovar). Excitation and emission wavelengths were set to 561 and 565–700 nm, respectively, to image mCherry-expressing bacterial populations. Each image covered an area of 197.91 197.91 μm (1156 × 1156 pixels) in the *XY* plane, with a 100 μm *Z*-stack acquired using the ZEN 3.6 Blue software (Zeiss). The exposure settings were optimized to ensure negligible autofluorescence from the background. Colony volume, sphericity, and fluorescence intensity were analyzed by using the Imaris Surface module with automatic thresholding (v10.1, Bitplane, Zurich, Switzerland).

The data were obtained through the IMARIS Imaging software (version 9.0, Bitplane) and went through a series of steps to extract meaningful insights. The data processing was implemented by using several Python libraries to facilitate reproducibility. Initially, CSV files generated by IMARIS were programmatically parsed using the csv and os libraries to extract relevant parameters, such as volume, sphericity, and mean intensity. These values were categorized and stored in separate Python lists and later transformed into pandas DataFrames for each experimental condition.^48^ Only bacterial colony volumes greater than 10 μm^3^ were selected for analysis, ensuring that the focus remained on the most significant data points. NumPy was used to implement a bootstrapping technique.^49^ This technique involved generating resampled data sets to estimate the distribution of colony volumes, sphericity, and intensity metrics.

Confidence intervals (95%) were then computed from the bootstrapped samples to understand the statistical variation across the conditions. To visualize the outcomes, Matplotlib^50^ and Seaborn^51^ were employed to generate comparative plots. These visualizations compare filtered data with bootstrapped confidence intervals, highlighting patterns and differences between different groups. The pipeline enabled the efficient processing of large-scale data sets and robust analysis of bacterial colony morphology and growth dynamics.

### Bacterial Encapsulation for calScreener-Based Isothermal Microcalorimetric Analysis

These experiments were performed using the calScreener Symcel isothermal microcalorimetry instrument (Catalog #1200001). For hydrogel preparation, 50 μL of polymer-bacteria suspensions was pipetted into individual wells of a sterile 48-well calPlate containing single-use sterile plastic inserts (SymCel, Sweden). The plate-containing inserts were then transferred to a UV chamber and exposed to UV radiation (365 nm) for 2 min at an intensity of 6 μW/cm^2^. After cross-linking, the insets with bacterial hydrogels were loaded into titanium vials and wells were closed to ensure optimal performance during high-precision calorimetric testing. After device calibration, calorimetric measurements were initiated as described in the literature.^52^ The data were analyzed using the calView 2.0 software. Heat flow data from the calScreener were evaluated based on key metrics such as maximum magnitude, peak timing, detection time, and area under the curve (AUC). These metrics were derived using Simpson’s rule with the assistance of the SciPy^53^ and Matplotlib libraries.

### Statistical Analysis

In Figure 4, the Mann–Whitney *U* test^54^ was used to assess pairwise differences between nine different formulations for each of the three parameters: volume, sphericity, and intensity mean. This nonparametric test was chosen because it compares ranks without assuming normality. Our data were found to be non-normally distributed upon visualization, which justified the use of nonparametric tests. Heatmaps of the pairwise *p* values between nine different formulations for sphericity, volume, and intensity mean are provided in the Supporting Information (Figures S2 and S3). The heatmaps were generated using the Seaborn^51^ and Matplotlib^50^ libraries in Python. In Figure 5, the Kruskal–Wallis Test followed by post hoc Mann–Whitney *U* test was performed to determine significant differences between the different conditions by testing the null hypothesis. The test was performed using the following online tool: https://www.statskingdom.com/kruskal-wallis-calculator.html.

## Results and Discussion

The hydrogels for bacterial encapsulation were made of Pluronic F-127 (Plu) and its diacrylated derivative (PluDA) mixed in three different ratios (1:0 = DA0, 1:1 = DA50, and 0:1 = DA100) at three different total polymer concentrations (15, 18, and 21 w/w%) to yield nine different formulations (Figure 1). Pluronic F-127 hydrogels undergo physical assembly at room temperature, and the diacrylated variants can be chemically cross-linked to increase gel stability and stiffness. Thus, the mechanical properties of these hydrogels are dictated purely by physical cross-linking (DA0), by physical and chemical cross-linking (DA50), or predominantly by chemical cross-linking (DA100). Notably, their stiffnesses ranged from a storage modulus of 3 to 110 kPa (Figure 1b).

**Figure 1:**
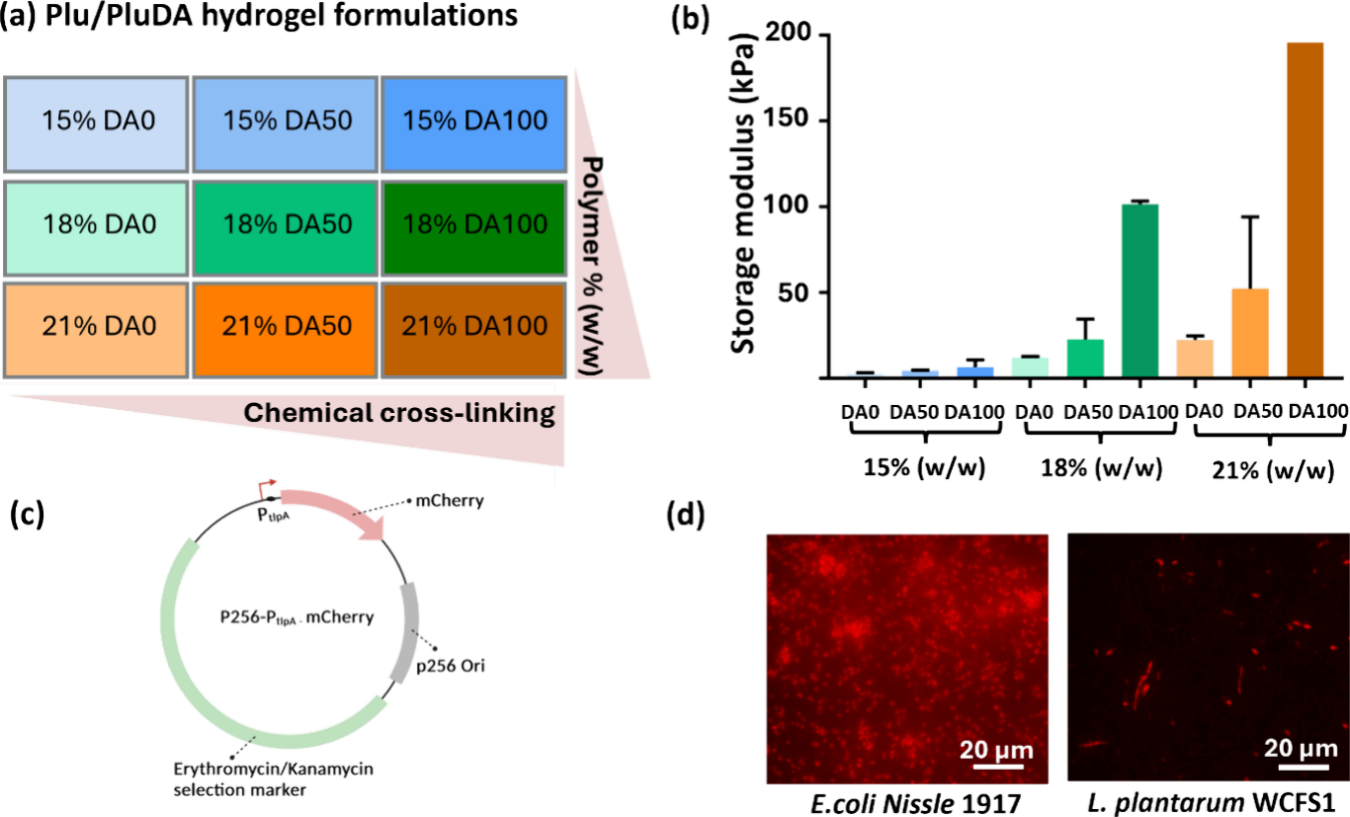
(a) Scheme representing the different combinations of Plu/PluDA in the hydrogels along with the colors used to represent these combinations throughout the study. DA 0 refers to 100% Plu, DA 50 refers to 50% Plu and 50% PluDA, and DA 100 refers to 100% Plu. (b) Bar graph representing the storage modulus of the nine combinations of Plu/PluDA hydrogels in the increasing order of polymer concentration and chemical cross-linking (*N* = 2). (c) Plasmid map for the plasmids with p256 origin of replication, P_tlpA_ promoter driving the expression mCherry fluorescent protein, and the selection marker being either kanamycin for E. coli Nissle or erythromycin for L. plantarum. (d) Fluorescence microscopic images of E. coli Nissle 1917 and L. plantarum WCFS1 expressing mCherry in liquid culture captured with the same microscopy settings.

The bacteria used in this study are fluorescent strains of E. coli Nissle 1917 and L. plantarum WCFS1 that were genetically engineered with the same plasmid coding for mCherry expression (Figure 1). This plasmid has a broad range replicon (p256) that is medium copy in E. coli (50–100) and low copy^3–5^ in L. plantarum. The expression of mCherry is driven by the P_tlpA_ promoter with high strength in E. coli^47^ and moderate strength in L. plantarum.^55^ The bacteria were mixed with the polymer solutions in a volume ratio of 1:9 at 4 °C to achieve a final bacterial density of OD_600 nm_ 0.01. For photopolymerization, the hydrogels were subjected to 2 min of exposure to 365 nm that has previously been shown to negligibly affect bacterial behavior.^13^

### Bacterial Behavior in Confinement at the Population Level

Bacterial growth and protein production in confinement are important functions in ELM that confer unique capabilities. The bacterial hydrogels were prepared with the polymers dissolved in the optimized media for each species (LB medium for E. coli and MRS for L. plantarum). For high-throughput analysis, the bacterial hydrogels were formed in the μ-Plate 96 Well 3D (Ibidi) within the lower well (10 μL) and covered with silicone oil to prevent evaporation of the water in the hydrogel (Figure 2a). Growth kinetics was measured 24 h after encapsulation using a microplate reader. The first interesting observation is that the overall growth profile was different for both encapsulated bacterial strains, although it is known that the doubling time of L. plantarum is more than that of E. coli. E. coli Nissle 1917 starts with a short lag phase for 2–4 h followed by an exponential phase for the next 6–10 h, and then a stationary phase is reached at around 12 h, most likely due to exhaustion of the available nutrients (Figure 2b). On the other hand, L. plantarum seemed to grow slower with a long lag phase for the first 10 h followed by two exponential phases with the second having a slower rate (<0.002 ΔOD_600 nm_/h) than the first (0.002–0.004 ΔOD_600 nm_/h) (Figure 2d). In comparison, when both strains were grown in well plates as nonencapsulated cultures, they exhibited a nearly negligible lag phase (<1 h) followed by two exponential phases (1–4 and 4–7 or 4–12 h) and a stationary phase (>7 or 12h) (Figure S1a,b). Notably, each phase was considerably shorter than what was observed with the encapsulated bacteria, indicating that mechanical confinement within the hydrogels slows bacterial growth. In the case of encapsulated E. coli, the sharp transition between the exponential and stationary phases could be a result of the bacteria growing as spatially confined colonies that exhaust the nutrients surrounding them and face diffusion limitations to support the entire colony.^56^ In the case of encapsulated L. plantarum, such sharp transitions between the two exponential phases are discernible in certain hydrogel formulations although less prominent possibly due to their slower and extended growth phases.^57^ It is to be noted that previous studies have reported that chemical cross-linking in Pluronic F127-based hydrogels does not significantly alter diffusion rates of small molecules within the matrix.^13^ Thus, it is not expected that differences in bacterial behavior are affected by differences in diffusion across different hydrogel compositions.

**Figure 2:**
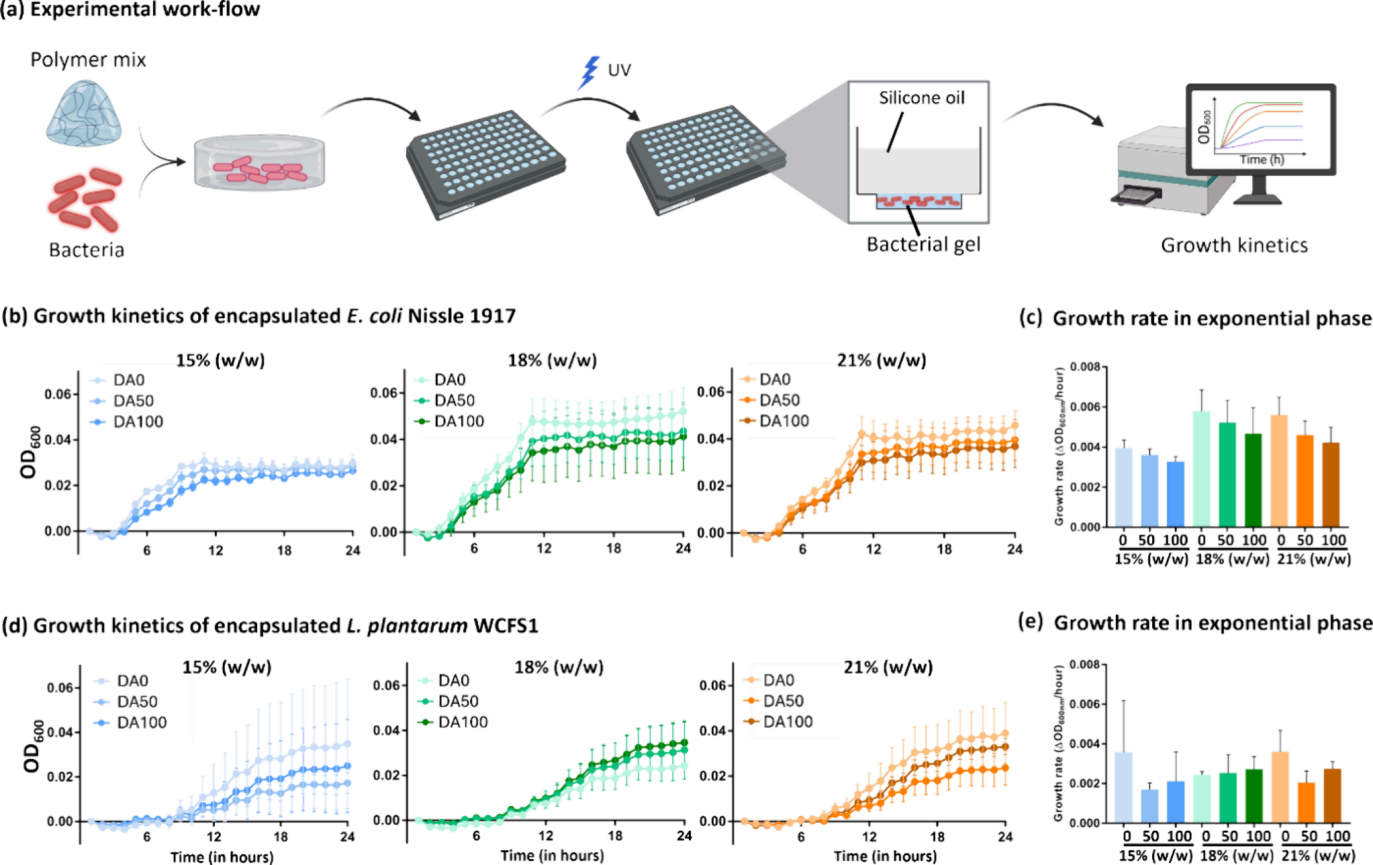
Experimental design and bacterial growth kinetics. (a) Schematic representation of the manual fabrication of bacterial hydrogels. Bacteria and polymer were mixed in a ratio of 1:10 followed by pipetting into the lower well of the μ-Plate 96 Well 3D (Ibidi) and photo-cross-linking of the bacterial gels and were set for growth kinetic measurements for 24 h. The zoomed-in section depicts the transverse section of a well with hydrogel (10 μL) in the bottom chamber covered with 50 μL of silicone oil. (b, d) Normalized growth kinetics of E. coli Nissle 1917 (b) and L. plantarum WCFS1 (d) expressing mCherry in the nine Plu/PluDA combinations for 24 h (*N* = 4). Normalization was done by subtracting the value at time zero from all the remaining time points. The data are presented as mean ± SD. (c, e) Column plots representing the exponential phase rate (change in OD_600 nm_ per hour) of E. coli Nissle 1917 (c) and L. plantarum WCFS1 (e) across nine polymer combinations between 4 and 9 h of growth for E. coli and 8 and 14 h of growth for L. plantarum (*N* = 4).

Within each polymer concentration, total growth and the rate of the exponential phase did not vary significantly from DA0 to DA100, although for E. coli, both parameters on average seemed to reduce with increasing degrees of chemical cross-linking (Figure 2c), while L. plantarum did not exhibit any apparent trend (Figure 2d). This could indicate that the mechanical differences between the different hydrogels possibly influence E. coli’s growth but not that of L. plantarum. A contributing factor to such a difference may be the higher turgor pressures in Gram-positive bacteria (>1000 kPa) compared to Gram-negative bacteria (30–300 kPa)^39^ since it is the primary force driving bacterial cell expansion.

Apart from monitoring bacterial growth, the intracellular production of mCherry protein was quantified by measuring the fluorescence intensity. Interestingly, protein production profiles were more aligned for both strains and seem to be associated with growth phases, with sequentially slow, rapid, and again slow production rates, although total production was an order of magnitude higher in E. coli compared to L. plantarum. In E. coli, the start of the rapid production phase (∼6 h) corresponded to the mid log phase of growth, but the end of the rapid phase (10–12 h) nearly correlated with the stationary phase of growth (Figure 3a). Contrastingly, in L. plantarum, the start of the rapid production phase (∼9 h) roughly correlates with the start of the first exponential phase, but the end of the first exponential phase (∼16 h) occurs a little beyond the mid rapid phase of production (Figure 3b). Interestingly, similar correlations between protein production phases and growth phases were observed in the nonencapsulated bacterial cultures (Figure S1a,b). This indicates that these are features of the promoter (P_tlpA_) in each strain, which are conserved even when the bacteria are encapsulated and exhibit slower growth and protein production rates.

**Figure 3:**
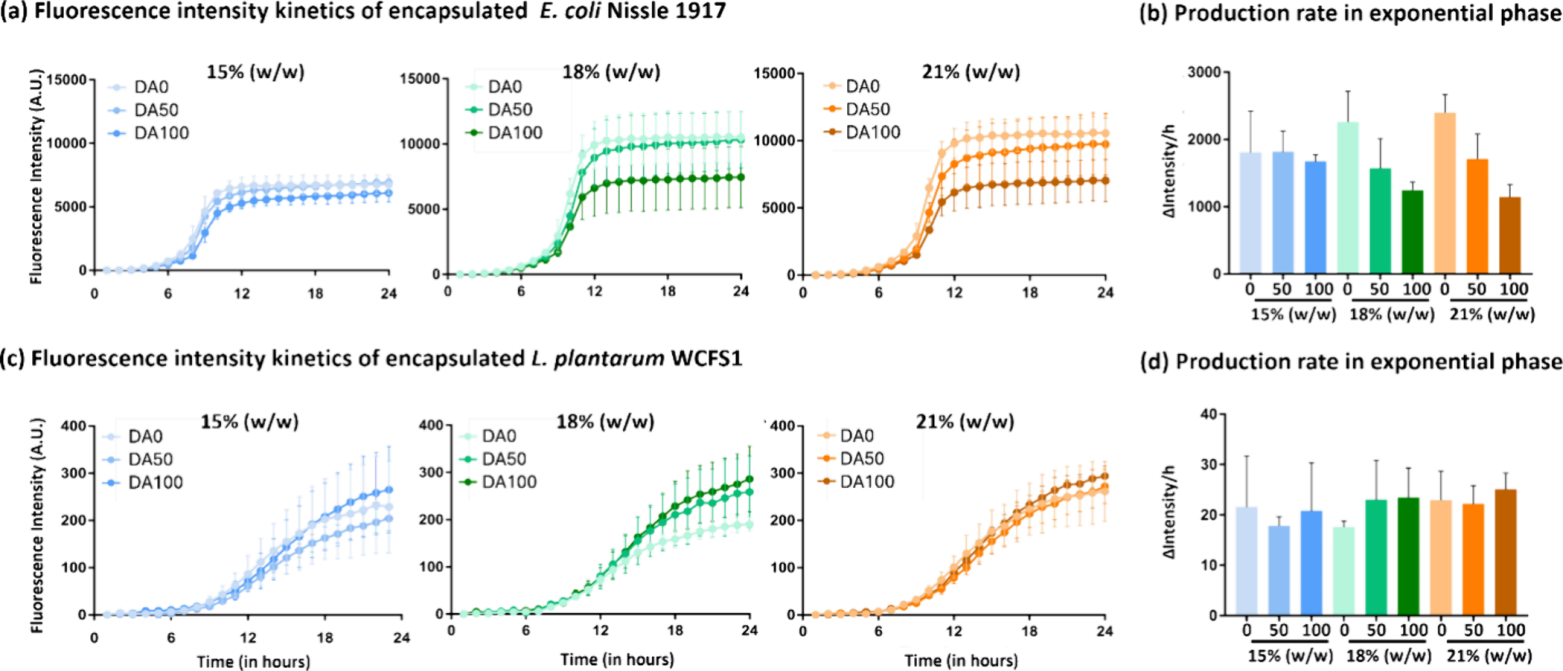
Protein production. (a, c) Normalized kinetics of fluorescence intensity increase in E. coli Nissle 1917 (a) and L. plantarum WCFS1 (c) expressing mCherry in the nine Plu/PluDA combinations for 24 h (*N* = 4). Normalization was done by subtracting the value at time zero from all the remaining time points. The data are represented as mean ± SD. (b, d) Column plots depicting the fluorescence intensity increase rate (change in fluorescence intensity per hour) of E. coli Nissle 1917 (b) and L. plantarum WCFS1 (d) expressing mCherry in the nine Plu/PluDA combinations between the 8 and 10 h for E. coli and 10 and 16 h for L. plantarum (*N* = 4).

With E. coli, for each polymer concentration, the overall protein production and rapid production rate seemed to reduce with increasing degrees of chemical cross-linking especially for 18 and 21% (w/w) formulations (Figure 3a,b). In contrast, no apparent trend was observed with L. plantarum. Thus, like with growth behavior, mechanical properties of the hydrogels seemed to influence protein production in E. coli but not in L. plantarum.

### Assessment of Growth and Protein Production in Bacterial Colonies

Within hydrogels, the bacteria are initially homogeneously distributed as single cells that grow into distinct colonies. After 24 h of growth, confocal microscopy was used to assess the sizes, morphology, and mCherry production levels of individual colonies. Notably, many more E. coli colonies were identified (900–1200) compared to L. plantarum (140 – 330) (Table S1), suggesting improved adaptation of individual E. coli cells to grow by overcoming mechanical restrictions in the hydrogels. It is to be noted that since both bacterial strains have different nutritional requirements, we used the MRS medium for L. plantarum and the LB medium for E. coli to ensure that both exhibit similar growth profiles (Figure S1a). Thus, the lower number of colonies observed in the L. plantarum hydrogels is most likely due to fewer cells adapting to the surrounding mechanical stresses and growing into colonies. This is further supported by the observation that the colony number in L. plantarum is the lowest in the most mechanically restrictive DA100 hydrogels. In terms of colony volumes, a wide range was observed in all hydrogel formulations, spanning 2 to 3 orders of magnitude. With E. coli, in all polymer concentrations, the distribution was the largest in DA0 and considerably smaller in DA50 and DA100, as has been observed in our previous study.^13^ While these distributions largely overlapped, significant differences of the means were found using the Mann–Whitney test, indicating a trend of decreasing colony volumes on average with higher degrees of chemical cross-linking (Figure 4b). On the other hand, for L. plantarum, the distribution of sizes did not seem to be influenced by the different degrees of chemical cross-linking, and a significant increase in mean colony volumes with increasing chemical cross-linking was seen in the 15 and 18% (w/w) polymer concentrations. While this is counterintuitive, an explanation could be that with greater mechanical restriction, fewer cells grew into colonies, and those that did were able to form large colonies on average. This is supported by the fact that the number of colonies in L. plantarum that could be measured decreased with increasing chemical cross-linking (Table S1).

**Figure 4:**
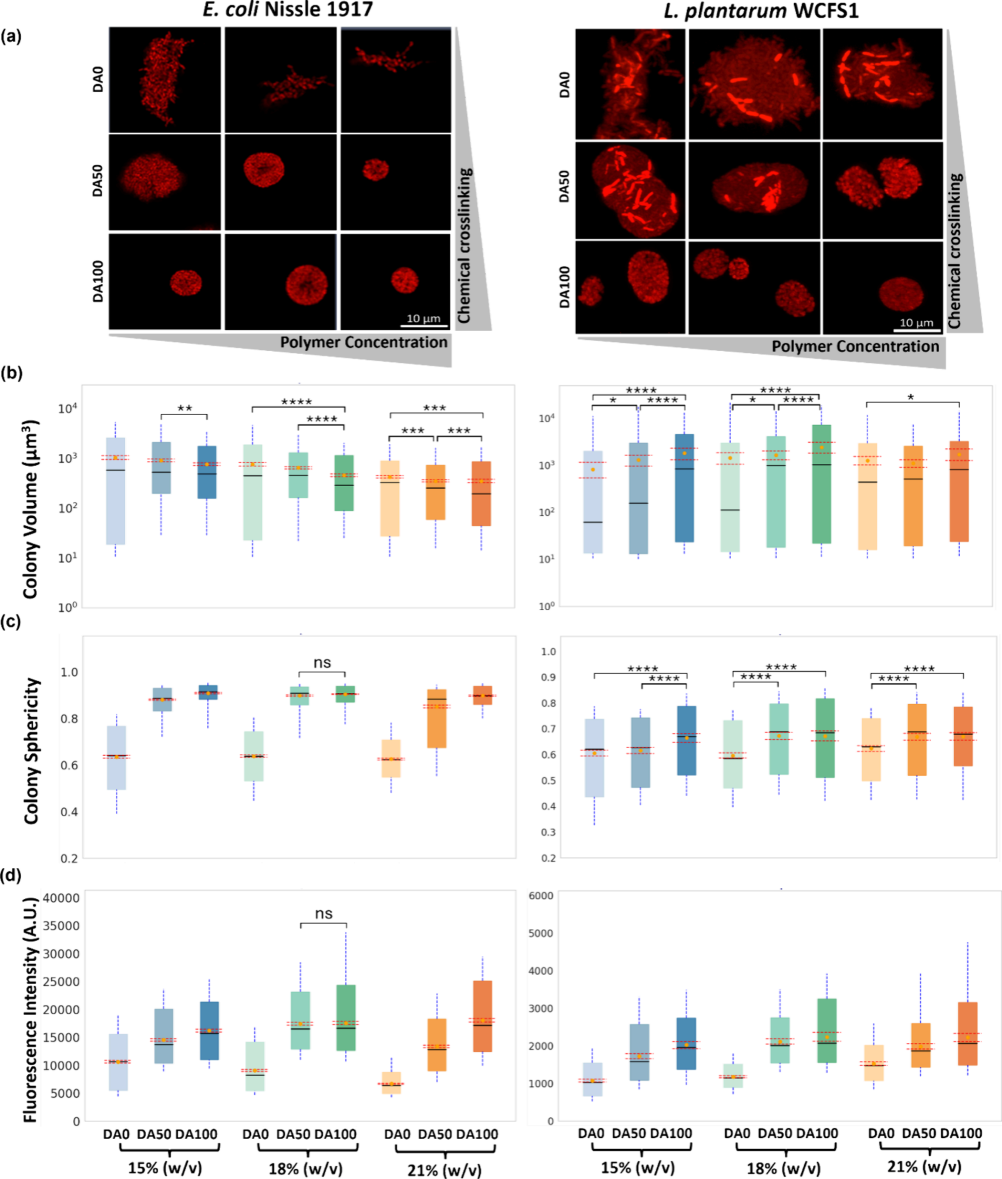
Confocal image analysis of bacterial colonies. (a) Confocal images of representative E. coli Nissle 1917 and L. plantarum WCFS1 colonies in the nine Plu/PluDA combinations after 24 h of growth. (b–d) Box plots representing the distribution of bacterial colony volume in cubic micrometer­(b), sphericity (c), and fluorescence intensity (d) after 24 h of growth in the different hydrogels. The graphs on the left represent E. coli Nissle 1917, and the ones on the right are L. plantarum WCFS1. The data were obtained from four individual samples, and distinct colonies above 10 μm^3^ were quantified. The Mann–Whitney *U* test was performed to determine significant differences (*p < 0.05, **p < 0.005, ***p < 0.0005, and ****p < 0.00005). In (c) E. coli and (d) both graphs, all distributions within each polymer concentration set were found to be significantly different (*p* < 0.0005), so only nonsignificant (ns) samples are marked. Comparisons across polymer concentration sets are not indicated (*N* = 4; boxes represent 25 and 75 percentile values, orange circles with dashed red lines represent the mean with confidence interval, and whiskers indicate the standard deviation).

Apart from affecting growth, mechanical restrictions within the hydrogels were expected to influence colony morphology, so we quantified colony sphericity on a scale of 0 to 1 (Figure 4c). In DA0 hydrogels with the least mechanical restrictions, both strains exhibited branch-like growth resulting in elongated colonies with low sphericity values (0.6–0.7). With E. coli, inclusion of chemical cross-linking caused the sphericity of the colonies to drastically increase with values >0.9. In contrast, with L. plantarum, the colonies were less branched but remained elongated in DA50 and DA100, resulting in only a slight increase in the sphericity. This indicates that in L. plantarum, there may be forces exerted between the cells within a colony capable of altering its geometry. This could be a consequence of intercellular interactions in L. plantarum that lead to autoaggregation mediated by adhesive proteins like aggregation promoting factor-D1 (APF-D1).^58^ While such autoaggregation has also been reported in E. coli Nissle 1917,^59^ it could be that this phenotype is not manifested under encapsulated conditions or the intercellular forces are not strong enough to alter the geometry of the colony.

Next, we assessed mCherry expression within the colonies to gain insights into the metabolic activity of encapsulated bacteria (Figure 4d). The first noticeable difference was that, with E. coli, the cells in a colony were relatively homogeneously fluorescent, while with L. plantarum, there was considerable heterogeneity. Such population-level differences in fluorescence profiles were also observed in the nonencapsulated bacterial cultures (Figure 1d), indicating that encapsulation did not alter this property. The next striking observation was that in both strains, the mean fluorescence intensity of the colonies increased with higher degrees of chemical cross-linking. Notably, for E. coli, this is in contradiction to the data in Figure 3a where DA100 shows a lower population-level fluorescence compared to DA0 and DA50 at 24 h. This suggests that even though the colonies exhibit, on average, higher fluorescence intensities with higher degrees of chemical cross-linking, the overall fluorescence intensity of the population is lower. However, it is to be noted that the microplate-based fluorescence spectroscopy method used to obtain the data in Figure 3a is more prone to scattering-induced variations in absolute values in turbid or heterogeneous samples compared to high-resolution confocal microscopy. Since, with E. coli, colony volumes, size distributions, and sphericity varied significantly with different degrees of chemical cross-linking, differential scattering could have influenced the absolute values of intensity measured using the microplate reader. As described in a previous study of ours,^13^ this trend with fluorescence intensity could be due to the mechanically restricted growth allowing the bacteria to dedicate more of its metabolic activity toward the production of the recombinant protein. However, this explanation may not completely justify the effect in L. plantarum since colony volumes are larger in DA50 and DA100. Rather, the growth of fewer colonies with higher degrees of chemical cross-linking may be a stronger contributing factor since the amount of nutrients per colony would be higher, enabling higher levels of recombinant protein production.

It is important to consider that along with the effect of mechanical properties, the slower growth rate of L. plantarum compared to that of E. coli is attributed to these phenotypical observations. Altogether, these analyses reveal that chemical cross-linking in these hydrogels more strongly influences colony growth in E. coli than L. plantarum, whereas recombinant protein production is similarly affected in both strains.

### Analysis of Metabolic Activity by Isothermal Microcalorimetric Analysis

To further investigate the impact of various hydrogel formulations on bacterial metabolism, we employed isothermal microcalorimetry (IMC) using a calScreener device (Symcel, Sweden). This technology enables real-time quantification of metabolic heat generated in bacterial hydrogels. For this, the bacterial hydrogels were formed in transparent plastic wells and placed in titanium vials, which were arranged in a 48-well format. For each strain, this allowed simultaneous assessment of all hydrogel formulations, nonencapsulated bacterial cultures, and blank medium in duplicates, along with two empty reference wells in each column. The titanium vials were set to maintain the temperature at precisely 37 °C; thus, metabolic heat was quantified as the heat removed to maintain this temperature. The resulting thermograms show real-time heat flow for 24 h, in which the total heat is the area under the curve. From liquid cultures, it is apparent that both organisms generate comparable amounts of heat (Figure 5a,b), although the heat is generated much more quickly in E. coli compared to L. plantarum. This indicates different rates at which the strains adapt to their environment and grow and is represented by the time taken for each organism to reach their first peak (Figure 5c). The time until the first peak represents the duration until the first metabolic transition occurs.^52,60,61^ Apart from the first peak, the thermograms also show multiple local maxima (other peaks) that indicate possible transitions in bacterial metabolism (e.g., exponential phase to stationary phase, aerobic to anaerobic, etc.). Here too, E. coli exhibits more peaks than L. plantarum (Figure 5d), suggesting its ability to switch to multiple different metabolic pathways as nutrients and oxygen become scarce.

**Figure 5:**
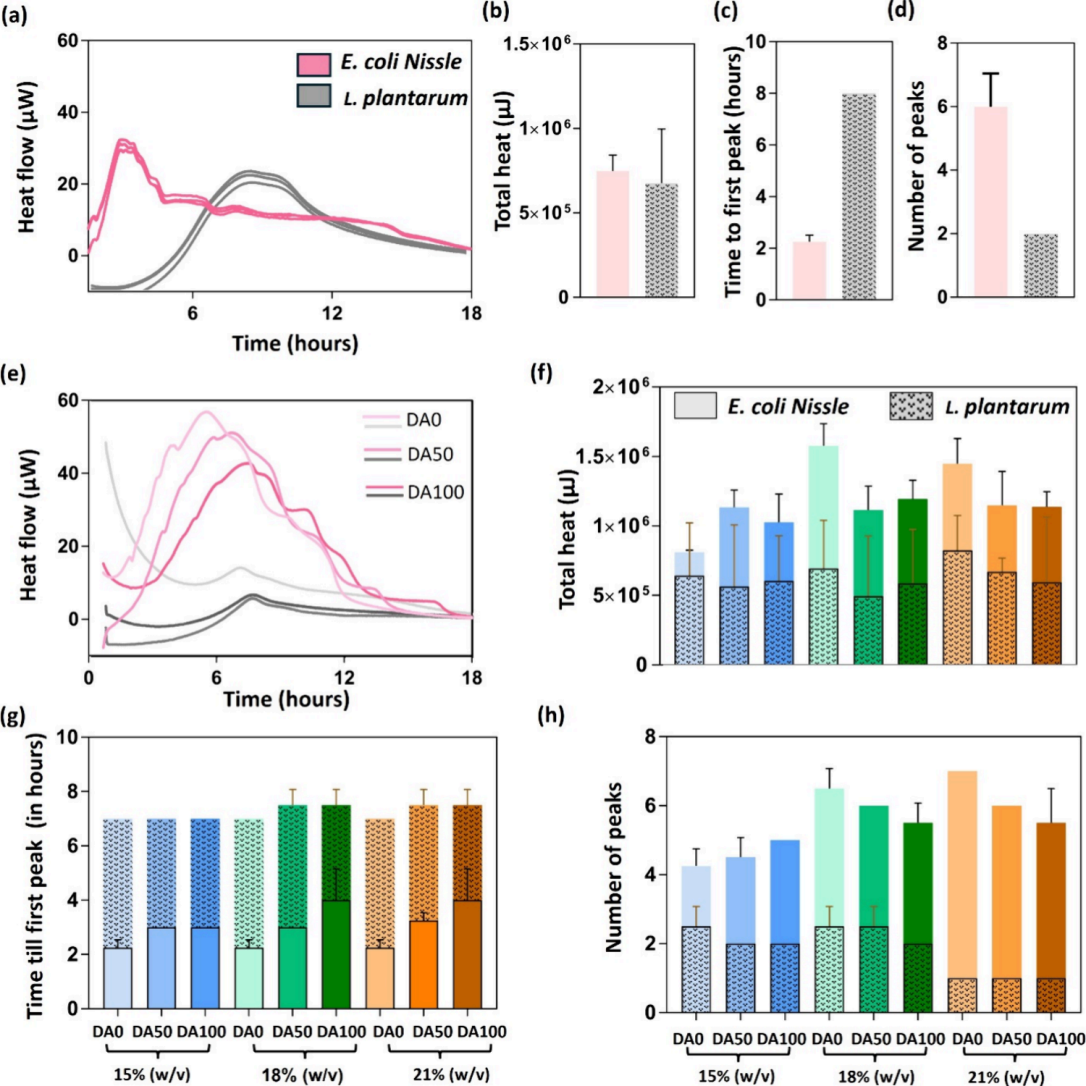
Isothermal microcalorimetry of encapsulated bacteria using calScreener. (a) Thermograms depicting heat flow (μW) quantified over time from liquid cultures of E. coli Nissle and L. plantarum incubated under isothermal conditions in the calScreener device at 37 °C for 24 h. (b–d) Bar graphs representing the total heat (b), number of peaks (c), and time to first peak (d) detected in liquid cultures of E. coli Nissle and L. plantarum. (e) Thermograms depicting heat flow (μW) quantified over time from encapsulated Plu/PluDA gels of E. coli Nissle and L. plantarum. (f–h) Bar graphs representing superimposed plots of the time to first peak (f), number of peaks (g), and total heat (μJ) (h) observed in thermograms of encapsulated Plu/PluDA gels of E. coli Nissle and L. plantarum (*N* = 4). All column plots represent mean ± SD.

Under encapsulated conditions, these thermograms are drastically altered. It is immediately apparent that the metabolic heat generated by E. coli is considerably higher than that of L. plantarum by a factor of 1.5–3 (Figure 5e). This indicates a higher metabolic rate in the E. coli population compared to L. plantarum and agrees with the results from Figure 2 showing faster growth of E. coli and Figure 3 in which more E. coli colonies were identified compared to L. plantarum. It also suggests possibly a more efficient and versatile use of the available nutrients by E. coli for energy production. Moreover, in E. coli, chemical cross-linking seems to influence total heat generation, whereas this does not seem to occur in L. plantarum (Figure 5f). For E. coli, in 18 and 21% (w/w) gels, those with chemical cross-links (DA50 and DA100) exhibit a significant drop in total heat compared with DA0, most likely due to more mechanically restricted growth. In the case of 15% (w/w), the lower total heat values could be attributed to the bacteria requiring less energy to overcome mechanical restrictions for growth. This is supported by the fact that the total heat of nonencapsulated E. coli growing in liquid culture is lower than or similar to that of the 15% (w/w) samples (Figure 5b,f). This suggests that at higher polymer concentrations, metabolic activity can be influenced by the extent of chemical cross-linking. In L. plantarum, the lack of significant differences across all hydrogel formulations aligns with the results in the previous sections, indicating minimal influence of the mechanical properties of the hydrogels on their growth. In agreement with this, nonencapsulated L. plantarum cultures also generate similar amounts of total heat compared to their encapsulated counterparts (Figure 5b,f).

In terms of the time to the first peak and number of peaks, once again, there is a clear difference between the two strains. In E. coli, the time to first peak occurs as soon as 2 h in the mechanically less restricted hydrogels and increases to 4 h with higher degrees of chemical cross-linking in the 18 and 21% (w/w) hydrogels (Figure 5g). In nonencapsulated liquid cultures, the time to first peak is 2 h for E. coli (Figure 5c). This suggests that E. coli is possibly adapting to nutrient limitations sooner than mechanical restrictions in the 15% (w/w) and DA0 hydrogels, while the slower growth in the more mechanically restricting hydrogels possibly delays the need for this adaptation.^13^ In L. plantarum, the time to first peak occurs almost uniformly around 7 h in all hydrogel formulations (Figure 5g) and nonencapsulated cultures (Figure 5c), which aligns with the observation throughout this study that mechanical restrictions within these hydrogels do not considerably influence growth and metabolic activity in this strain. Beyond the first peak, E. coli exhibits four to seven peaks in total, while L. plantarum has one to three (Figure 5h). This suggests that E. coli is able to adapt to the mechanical restrictions and, eventually, nutrition limitations by switching between different metabolic pathways, whereas L. plantarum does not have this versatility.^62,63^ In E. coli, this is further supported by the fact that more peaks are observed in 18 and 21% (w/w) hydrogels. However, chemical cross-linking in these hydrogels reduces the number of peaks by at least one, suggesting that a mechanical barrier is probably reached beyond which further growth cannot be achieved through metabolic changes. The only parameter that chemical cross-linking seems to considerably affect in L. plantarum seems to be recombinant protein production levels in colonies as observed in Figure 4d, but the IMC analysis reveals that this is not due to any major shift in overall metabolic activity. Therefore, the difference is possibly only due to improved translational efficiency and an efficient realignment of resources toward recombinant protein production when colony growth or numbers are lower.^64,65^

## Conclusions

This study provides a comprehensive comparison of the adaptive responses of probiotic Gram-negative E. coli Nissle 1917 and Gram-positive L. plantarum WCFS1 to mechanical confinement within hydrogel matrices. Both bacterial species have been found to form biofilms within the human body. Assuming that the mechanical properties of these native biofilms are similar to those of bacterial biofilms that have been extensively studied,^35–38^ they are expected to be comparable with the ELMs in this study. Our findings reveal that while both bacterial species can grow and produce recombinant proteins under confinement, their responses to the mechanical properties of the encapsulating material differ significantly. Time-resolved analysis of growth and recombinant protein production showed that encapsulation slows down and delays the different phases in both bacteria, with L. plantarum being natively slower in both parameters. However, the correlations between protein production and growth phases seem to be conserved, revealing that encapsulation does not alter the performance of the promoter in both bacteria (Figure 6). As shown in Figure 6, increasing chemical cross-linking led to opposite responses in encapsulated E. coli and L. plantarum, whereas fluorescent protein production and total metabolic heat followed similar trends. Through confocal microscopy analysis, we observed that E. coli exhibited notable changes in colony growth, size, morphology, and metabolic activity in response to varying hydrogel stiffness, indicating greater sensitivity to mechanical constraints. In contrast, L. plantarum showed a more robust growth pattern with minimal changes in colony morphology and metabolic activity, likely due to its thicker cell wall and slower growth rate, which may confer an advantage in overcoming mechanical restrictions. However, colonies of both bacteria exhibited increased recombinant protein production with a higher matrix stiffness, which underscores the potential of optimizing hydrogel properties to enhance the functionality of ELMs. These insights into species-specific adaptations to mechanical confinement advance our understanding of the interplay between material properties and microbial physiology. This knowledge is crucial for the rational design of ELMs tailored for therapeutic applications where precise control over the behavior of engineered probiotic bacteria is essential. Overall, this study highlights the importance of considering the mechanical environment in the development of ELMs and provides a foundation for future research aimed at optimizing material–microbe interactions for various applications.

**Figure 6:**
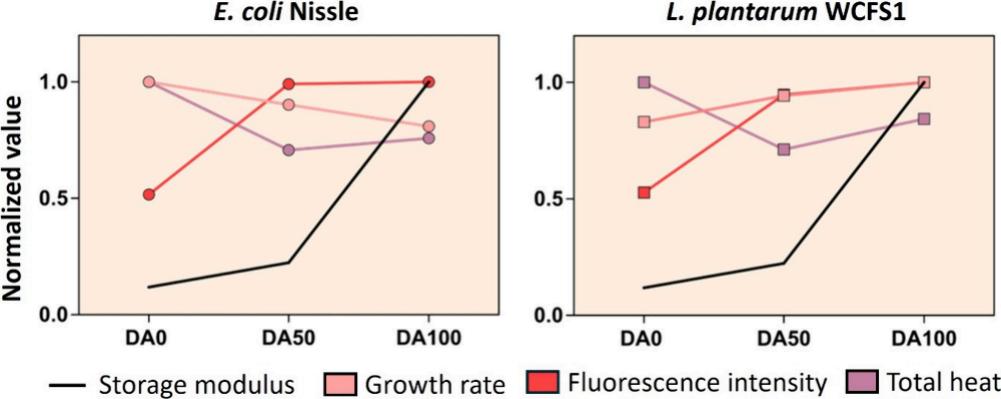
Summarized plots of E. coli Nissle and L. plantarum WCFS1 representing the common trends observed in encapsulated formats of the respective strains expressing mCherry fluorescent protein in 18% w/w Plu/PluDA gels. Normalized values were obtained by dividing the respective data points with the highest magnitude within a data set.

## Supplementary Material



## Data Availability

The information provided in the main text and Supporting Information are sufficient to reproduce the experiments described in this study. The raw and processed data sets along with relevant metadata are available from the corresponding author upon reasonable request.

## Notes

Bacterial growth kinetics in liquid culture and microscopic images of engineered strains (Figure S1); heatmaps representing the statistical significance in terms of *p* values obtained using the Mann–Whitney *U* test (Figures S2 and S3); bacterial viability assay results (Figure S4a); and flow cytometry of bacterial populations stained with live/dead staining (Figure S4b) (PDF)
