# Supplementary material for: Adaptations of Gram-Negative and Gram-Positive Probiotic Bacteria in Engineered Living Materials
Source: ACS Biomater Sci Eng. 2025 May 13;11(6):3773–84. doi: 10.1021/acsbiomaterials.5c00325 (PMC12152833; doi:10.1021/acsbiomaterials.5c00325)
Supplement: Supplementary file 1 [file ab5c00325_si_001.pdf]

SUPPORTING INFORMATION

Adaptations of gram-negative and gram-positive probiotic bacteria in engineered living materials

Varun Sai Tadimarri[1,4], Tanya Amit Tyagi[1,4], Cao Nguyen Duong[1], Sari Rasheed[2,4], Rolf Müller[2,3,4], Shrikrishnan Sankaran[1]

[1] INM - Leibniz Institute for New Materials, Saarland University, Campus D2 2, 66123 Saarbrücken, Germany

[2] Helmholtz Institute for Pharmaceutical Research Saarland (HIPS), Helmholtz Center for Infection Research (HZI), Saarland University, Campus E8 1, 66123 Saarbrücken, Germany

[3] German Centre for Infection Research (DZIF), Partner Site Hannover, Braunschweig, Germany

[4] Saarland University, 66123 Saarbrücken, Germany

*E-Mail: Shrikrishnan.sankaran@leibniz-inm.de

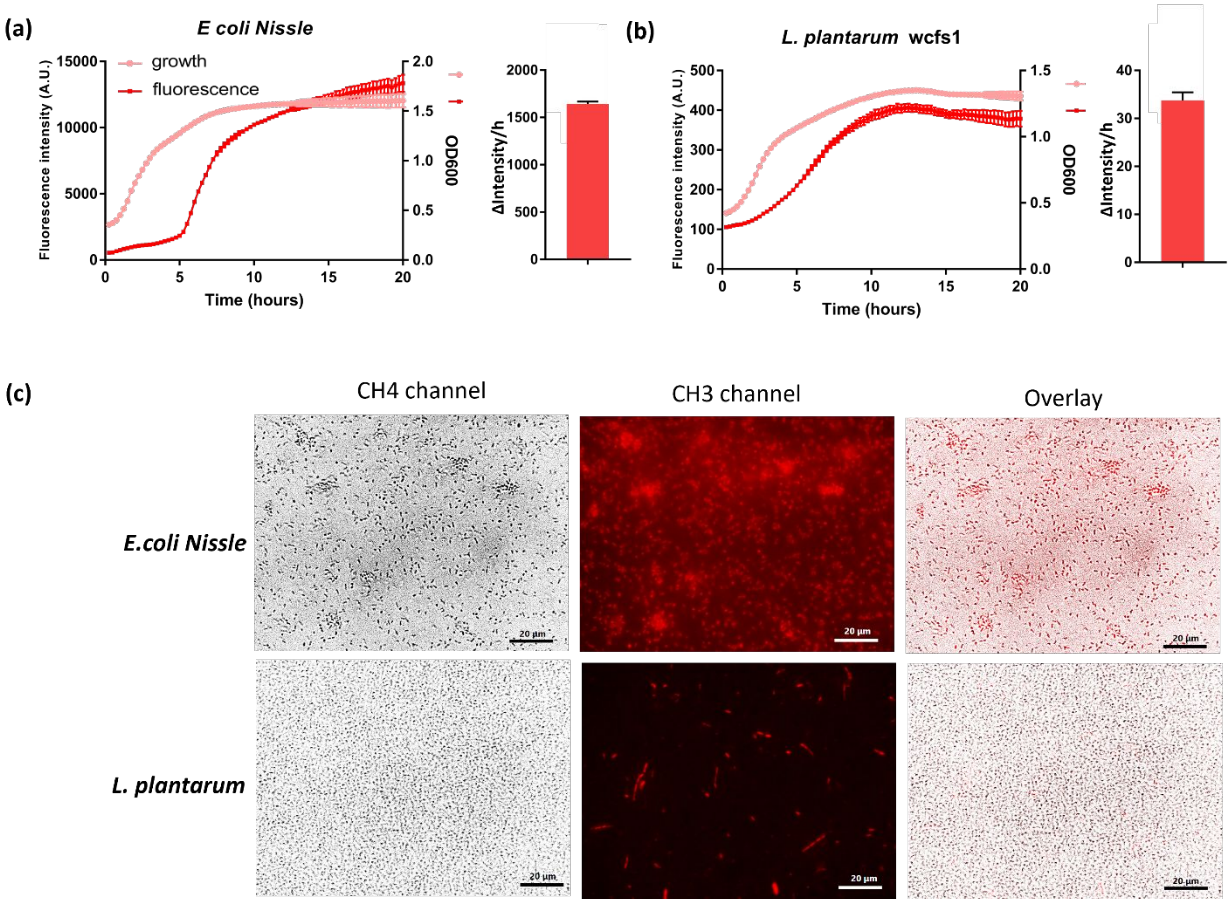

Figure S1: Bacterial growth kinetics: *Plots showing the growth kinetics and fluorescence expression kinetics of liquid cultures of* ***(a)*** *E. coli Nissle expressing mCherry, and* ***(b)*** *L. plantarum expressing mCherry. The bar graphs represent the fluorescence intensity rate during the exponential phase, between 5 – 10 hours of growth kinetics.* ***(c)*** *Microscopic images from cultures of E. coli Nissle and L. plantarum expressing mCherry after 20 hours of growth kinetics.*

Poly. conc. (w/w)	Chem. cross-link.	*E. coli*	*L. plantarum*
**15%**	DA0	1014	333
	DA50	1249	307
	DA100	1174	143
**18%**	DA0	1102	314
	DA50	992	203
	DA100	1033	142
**21%**	DA0	1237	246
	DA50	904	236
	DA100	832	169

Table S1: *List of number of colonies analysed per condition of E.coli and L. plantarum to determine bacterial colony volume, colony sphericity and fluorescence intensity mean.*

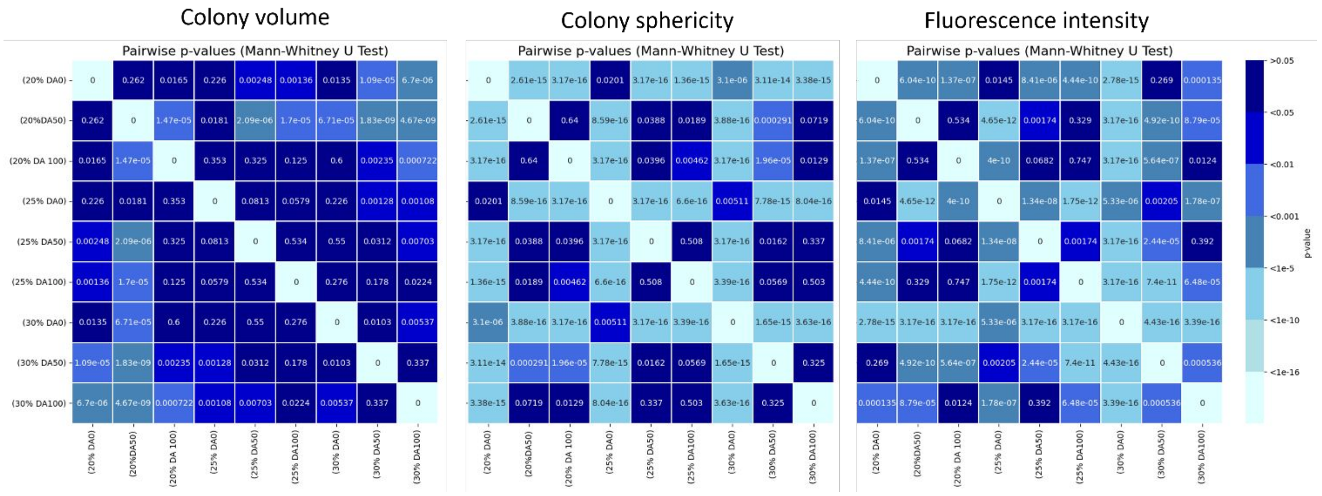

Figure S2: *Heatmaps representing the statistical significance in terms of P-values obtained using Mann-Whitney U test, to assess pairwise differences between nine different formulations for each of the three parameters assessed for E. coli Nissle: Colony volume, Colony sphericity and Fluorescence intensity.*

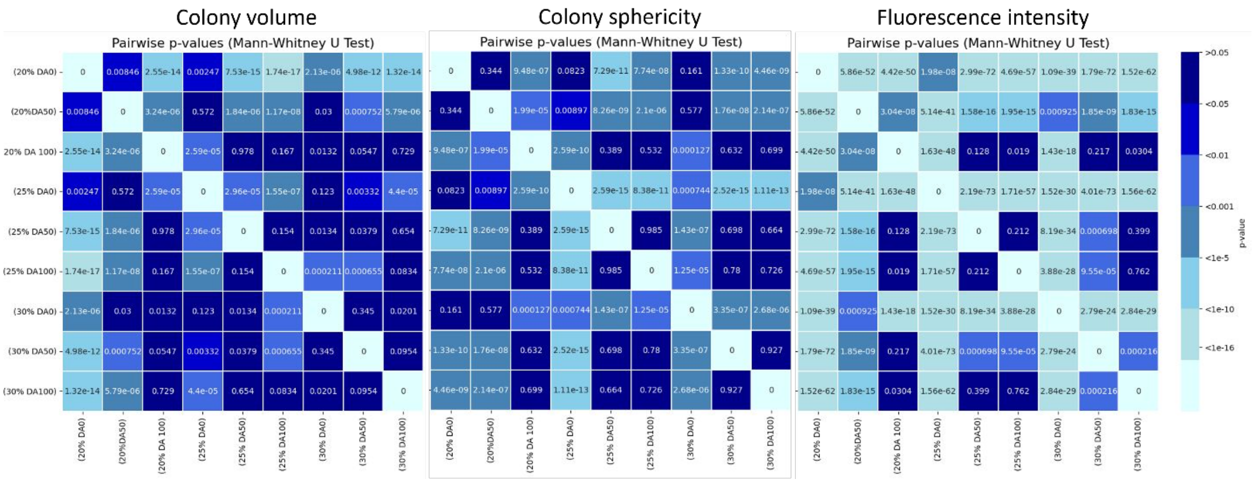

Figure S3: *Heatmaps representing the statistical significance in terms of P-values obtained using Mann-Whitney U test, to assess pairwise differences between nine different formulations for each of the three parameters assessed for L. plantarum: Colony volume, Colony sphericity and Fluorescence intensity.*

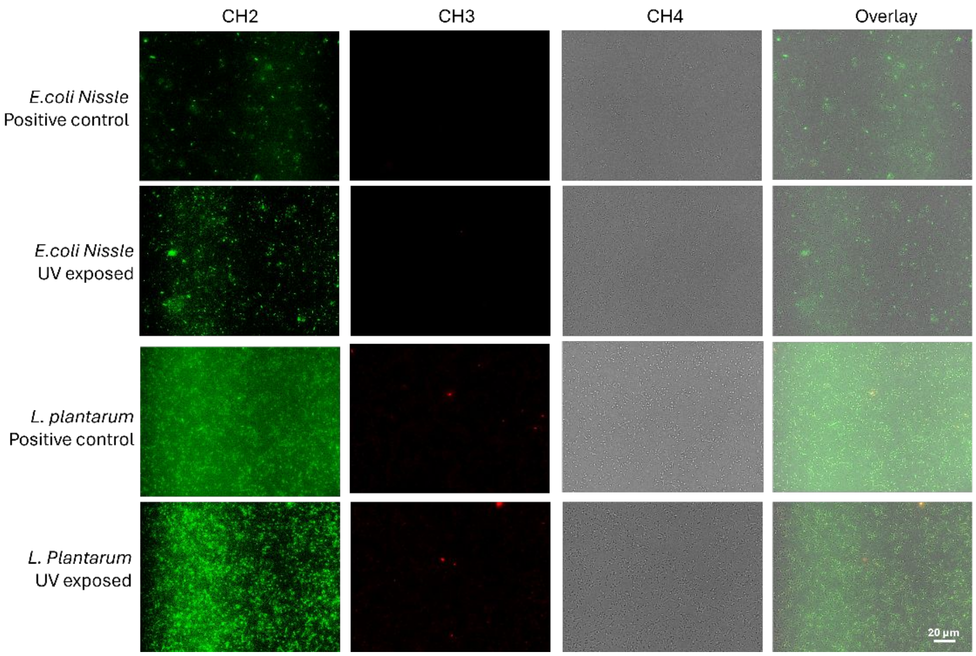

Figure S4a: Bacterial viability assay (Live/dead staining): *Fluorescence microscopic images of E. coli Nissle and L. plantarum stained with live/dead Baclight viability stain. CH2 channel represents the live bacteria, with green fluorescence of SYTO-9 dye. CH3 channel represents the dead bacteria, with red fluorescence of propidium iodide dye. CH4 represents the brightfield channel and the last lane is the overlay of all three channels.*

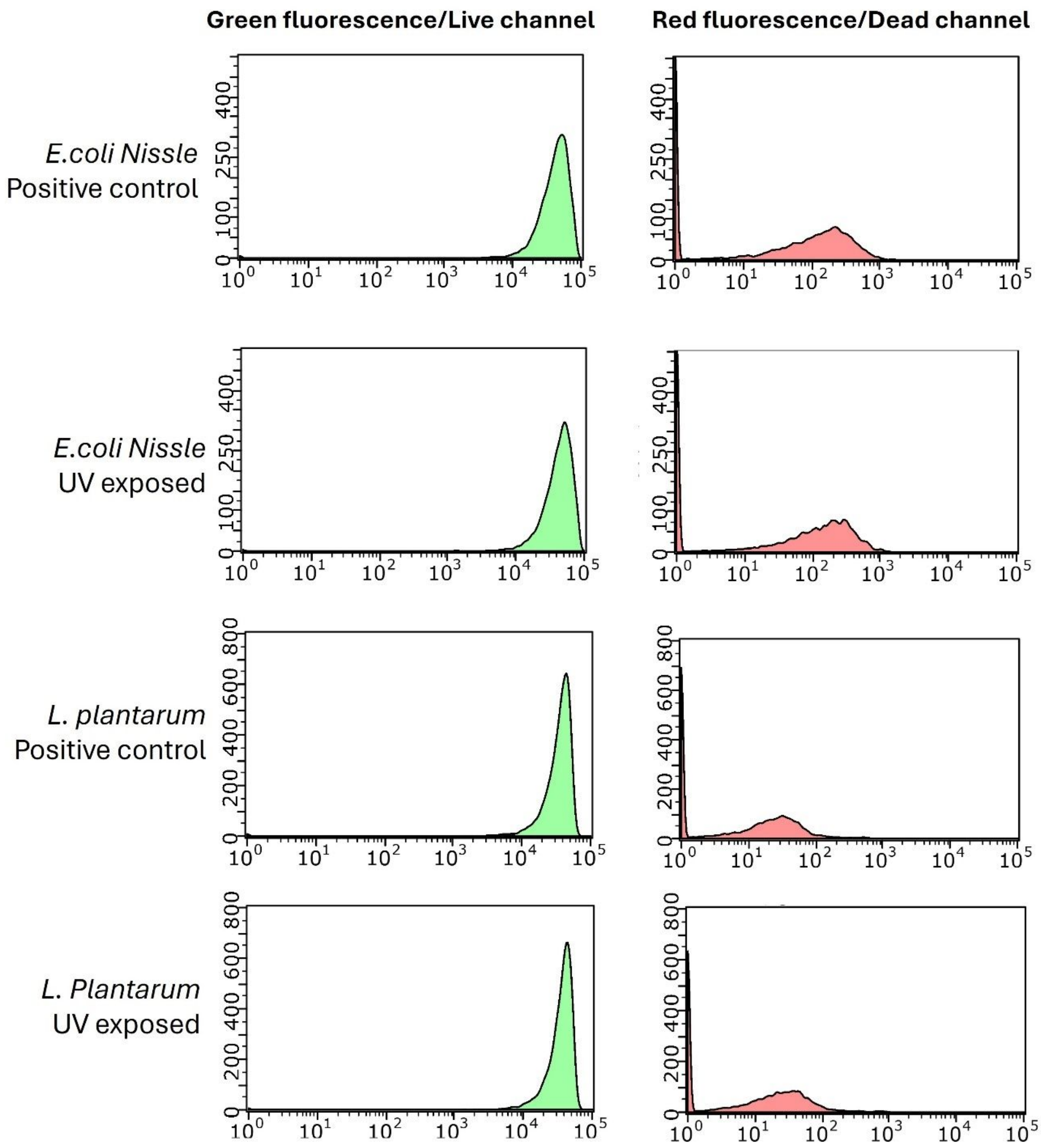

Figure S4b: Flow cytometry: *Plots representing the bacterial populations in green and red channels after live/dead staining. The first row represents the data of E. coli Nissle's positive control (untreated/live bacteria), the second row represents the population distribution of E. coli Nissle after exposure to UV for 2 minutes, the third row represents the data of L. plantarum's positive control (untreated/live bacteria), and the fourth row represents the population distribution of L. plantarum after exposure to UV for 2 minutes.*
